# Application of Fiber Bragg Grating Sensor Technology to Leak Detection and Monitoring in Diaphragm Wall Joints: A Field Study

**DOI:** 10.3390/s21020441

**Published:** 2021-01-09

**Authors:** Yapeng Zhang, Congxin Chen, Yun Zheng, Yong Shao, Chaoyi Sun

**Affiliations:** 1 State Key Laboratory of Geomechanics and Geotechnical Engineering, Institute of Rock and Soil Mechanics, Chinese Academy of Sciences, Wuhan 430071, China; zhangyapeng17@mails.ucas.edu.cn (Y.Z.); cxchen@whrsm.ac.cn (C.C.); shaoyong19@mails.ucas.ac.cn (Y.S.); cysun@whrsm.ac.cn (C.S.); 2 University of Chinese Academy of Sciences, Beijing 100049, China

**Keywords:** diaphragm wall, joint, leakage detection, fiber Bragg grating sensor, field study

## Abstract

Joints between diaphragm wall panels are weak spots in wall construction. It is essential that potential leak sites are detected prior to excavation. In this study, a novel leak detection and monitoring system is presented that is based on fiber Bragg grating (FBG) sensing technology. A field study was performed in a deep excavation supported by diaphragm walls (in Hohhot, China) to validate the feasibility and effectiveness of the proposed method. Two schemes were trialed; one using pipes made of stainless steel, and one used a pipeless method. The results of the field study are presented and discussed. They show that potential leak sites in the wall joints could be determined prior to excavation using the proposed detection method. Stainless steel is a good material to use to make the detection tube because it can protect the FBG sensors and heating belts from damage and is more sensitive to water leakage. The field study provides good evidence for the feasibility of the new detection system. It also provides valuable experience for the field application of the system and has generated useful data to use in follow-up work.

## 1. Introduction

Diaphragm walls (D-walls) are commonly used in deep underground construction projects—e.g., deep basements for high-rise buildings, underground railway stations, underground parking garages, and other underground facilities that require deep excavations. They act as retaining walls to resist the lateral pressure acting on their backs and also obstruct the flow of water, thus acting as a waterproof curtain to ensure that the deep excavations remain dry.

D-walls are constructed using a method based on the advances made in ‘slurry wall’ technology and, in most cases, they perform well. However, D-walls consist of individual panels that are cast piece-wise and separated by watertight joints [1]. As a result, water and soil particles may pass through these joints into the pit if they contain defects. Different methods have been applied on construction sites in order to improve the watertightness of the wall joints—e.g., installing rubber bands, metal sheets, or precast concrete elements as water-stops on the ends of the panels of the D-wall [2]. However, the construction procedures involved are complex and include trench excavation, refreshing of the bentonite suspension, lowering of steel reinforcement cages, installation of stop ends, and concreting. Such procedures may also increase the risk of imperfections occurring. For example, water-stops may be missed or damaged, concrete surfaces may not be thoroughly clean, there may be delays in the refreshing of the bentonite, and other problems may occur with the joints between the neighboring panels during the construction of the D-wall [3]. As a result, it is difficult to control the watertightness of the joints during the construction work [4,5]. 

When a joint in a D-wall does contain defects, leakage may occur. If the excavation is below the groundwater table, there is the possibility that water inrush and piping may occur during excavation, resulting in land subsidence and even disaster [6,7,8,9,10]. Furthermore, the bearing capacity of the foundation will decrease with the inrush of groundwater into the excavation zone [11]. Previous studies and historical precedent show that severe damage can be caused to the buildings (or other structures) nearby due to leakage occurring in generic joints between adjacent D-wall panels [3,12,13]. Consequently, the risks associated with leakage must be taken seriously, and detection and monitoring activities must be implemented to find potential leakage sites. Thereafter, a repair program can be put in place to prevent the situation from getting worse.

Most of the time, leakage problems are addressed by carrying out visual inspections of the leakage points in the D-wall joints. Naturally, this requires the joints to be exposed after excavation. However, the situation may worsen (with, for example, the inrush of water) by the time that happens. Furthermore, the repair program may be very difficult and the costs very high under the circumstances [14]. Therefore, an ability to obtain reliable information about the state of the D-wall joints prior to excavation is highly desirable. Appropriate and timely mitigating measures can then be performed based on such information (i.e., the severity and locations of potential leakage sites). In this manner, the risk of leakage occurring during excavation can be controlled.

Several methods have been suggested for detecting leaks in the joints between adjacent panels in D-walls in advance of excavation. In general, these methods can be classified as either geophysical or hydrogeological, depending on their operating principle [15]. For example, cross-hole sonic logging has been successfully implemented in several projects to determine the quality of joints and locate local defects in D-walls [4,16]. However, in this method, access tubes need to be installed on both sides of the joint to accommodate an acoustic source and receiver, respectively. Furthermore, the source and receiver need to be level with each other and be pulled up simultaneously. This complicates the detection process and requires the use of skilled technicians [2]. 

Hydrogeological methods detect potential leakage points in D-walls by observing water level recovery during field pumping tests [3,7]. Unfortunately, the locations of any leaks cannot be determined using this method. Furthermore, when bentonite inclusions are employed to produce high hydraulic resistance in the joints, any potential weak spots in the wall will not be found in the pumping tests prior to excavation [16]. 

In recent years, fiber-optic-distributed temperature sensor technology (FODTS) based on Brillouin and Raman scattering, and quasi-distributed temperature sensor technology based on fiber Bragg grating (FBG), have developed rapidly and been employed to perform health monitoring for civil structural works, such as leakage detection of dam and petroleum pipeline [17,18,19,20,21,22,23,24]. With the advanced sensor technology, innovative methods have also been devised to detect and localize potential leakages in D-walls based on FODTS technology [5,16,25,26]. However, when such testing is carried out during the construction of a D-wall, the test procedure is often interrupted by some other aspect of the construction process (e.g., bentonite refreshing, concrete casting, etc.). Moreover, FODTS is insensitive to small temperature variations and susceptible to environmental temperature in the case of small seepage [25,27]. Few publications are available regarding leakage detection in D-wall joints using FBG sensing technology. Very recently, Zheng et al. proposed a new method to detect leaks in D-wall joints [15]. Unlike the previous studies of leak detection in D-wall panels using FODTS [5,16,25,26], FBG sensors are used to detect the potential leakages in the D-wall joints in this work. Active heating technology is also used to increase the signal-to-noise ratio of the proposed system. The feasibility of the method has been verified using a series of physical model tests which showed that the position and time of occurrence of leakage could be determined in a laboratory setting [15]. However, the applicability of the method has not yet been reported in the field.

Therefore, the purposes of this study are to detect potential leakage sites and verify the feasibility of the aforementioned ‘leakage detection system for D-wall joints’ (LDS-DWJ) in a practical, real-life situation. A deep excavation in Huhhot in China was selected, in which to carry out our field study. Details of the installation and operation of the detection system as applied to this project are presented herein. Specific issues encountered during the field study and the solutions implemented to overcome them are also demonstrated. Finally, the test results and feasibility of the method are discussed in detail. The experience and data accumulated during the field application are very valuable and should help improve the method in follow-up work.

## 2. Detection Method and Established System

The LDS-DWJ discussed in this paper is based on FBG thermal sensing technology. The system monitors the temperature distribution (produced using active heating) inside the wall joints using FBG sensors. Temperature tracing is then used to determine the state of the D-wall joints and gather detailed information about the potential leakage occurring there.

### 2.1. System Configuration

**Figure 1:**
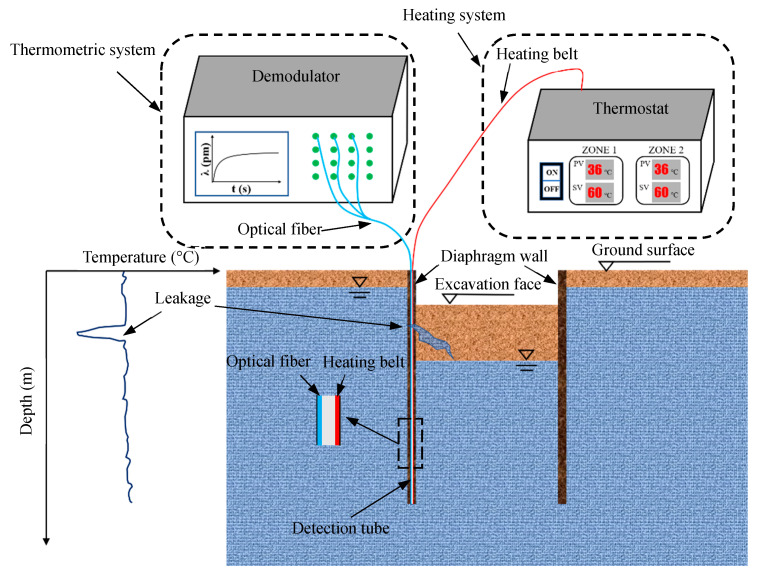
Diagram illustrating the leakage detection system for diaphragm wall joints (LDS-DWJ). The temperature measured using the fiber Bragg grating (FBG) sensors fluctuates at the point where the fiber encounters water leakage (modified from Zheng et al., 2020 [15]).

The basic configuration of the LDS-DWJ is depicted in Figure 1. The system consists of three subsystems: detection tube, heating system, and thermometric equipment.

The detection tube is positioned vertically along the D-wall joint. The tube itself is made of stainless steel, with diameters ranging from 2 to 5 cm, depending on practical application. The detection tube has four functions: (i) to act as a waterproof barrier, (ii) to conduct heat, (iii) to house the optical fibers and heating cables, and (iv) to protect the components within.

The heating subsystem is composed of a heating belt and a thermostat. The heating belt is fixed firmly onto the inner surface of the detection tube and is used to increase the temperature of the detection tube to a preset value, as determined by the thermostat (i.e., ‘active heating’ is employed).

The thermometric subsystem is made up of an optical fiber (containing a series of FBG sensors) and an FBG demodulator. It is used to monitor the temperature along the D-wall joints. The optical fiber is also fixed onto the inner surface of the detection tube. A narrow range of wavelengths is reflected from a sensor when it is illuminated by broadband light from the demodulator. The light reflected is sent to the demodulator device, which determines the temperature along the length of the detection tube, thus tracking the temperature of the wall joint [26,28,29].

### 2.2. Detection Method

Figure 1 summarizes the detection method and operating principle of the detection system. One of the main jobs undertaken in the detection process is to artificially elevate the temperature field of the detection tube to a preset value using the electrical heating belts. The preset value is determined depending on the surrounding temperature. In other words, the preset value should be higher if the surrounding temperature is high. As a result, significant temperature difference between the detection tube and its surroundings is generated, which can improve the sensitivity of the instruments to temperature variation.

The temperature field of the detection tube is then simultaneously monitored using the FBG sensors from the top of the tube to the bottom. If there is a leak, the temperature of the detection tube at that point is reduced because the leaking water absorbs some of the thermal energy from the pipe at that location. Accordingly, the signal captured by the FBG sensors can be processed to determine whether or not there is water leakage.

Zheng et al. [15] have described the detection system and its operating principles in some detail and readers can refer to that paper for further information. The paper also demonstrates that the novel detection system can be used to effectively determine the location and severity of leaks in laboratory test models. However, there are further issues that need to be resolved when the method is used in practical situations. For example, engineers need to decide exactly how the FBG sensors and heating belt will be installed, how the long detection tube should be spliced and connected with the steel cage, how the cables will be protected when the steel reinforcement cage is lowered, exactly how the detection process should be implemented, and so on. To help solve these problems, a deep excavation project was selected for field study.

## 3. In Situ Application of the New Detection System

### 3.1. Project Description

**Figure 2:**
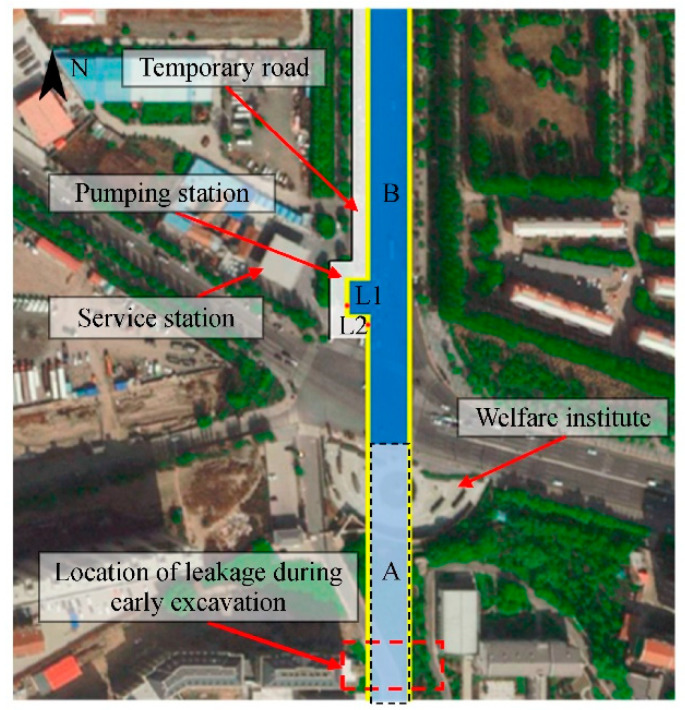
A plan view of the open pit excavation part of the project and layout of the instrumentation.

A new urban expressway is being constructed in Hohhot in northern China (111°43′35″ E, 40°45′52″ N). Part of this project involves the construction of a 770-m long underground tunnel, as shown in Figure 2. The deep excavation required was carried out using the ‘cut-and-cover’ method.

**Figure 3:**
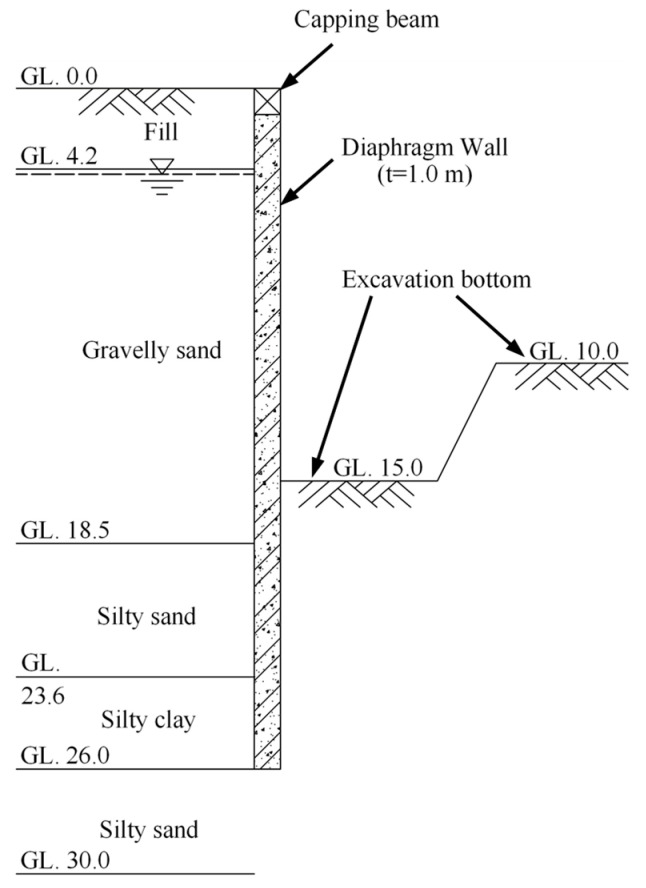
Cross-section and geological profile of the excavation at the pumping station. It should be noted that the use of struts is not considered in this paper and so they are not shown here for the sake of simplicity. (Units: m.).

**Figure 4:**
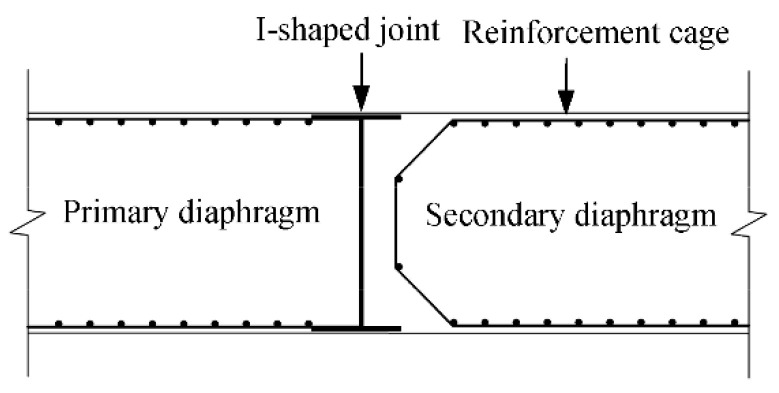
Geometry of the I-shaped sheets used as joints between adjacent diaphragm wall (D-wall) panels (top view).

The LDS-DWJ was applied to the D-walls constructed in this part of the excavation project. Figure 3 presents a schematic diagram of a typical cross-section of the deep excavation in the area of the pumping station. The maximum depth of the excavation in this area is 15 m (10 m in the area outside the pumping station). The excavation is 37.6 m wide near the pumping station, while it is 30.6 m in the other areas. The excavation was protected by installing multi-propped D-walls to a depth of 26 m. These are generally 1000 mm thick, and are intended to act as a waterproof curtain to facilitate dry conditions in the deep excavation. The length of a single wall panel ranges from 3.3 to 6.0 m, depending on the precise circumstances encountered. Adjacent panels were joined together using I-shaped metal sheets (Figure 4).

Figure 3 illustrates the soil profile and groundwater conditions encountered at the pumping station site. As can be seen, there are four layers encountered from the ground surface to the bottom of the D-wall. The surface is essentially a layer of backfilled artificial earth. The following two layers are gravelly and silty sand with high permeabilities. The bottom layer is made of silty clay. According to the hydrogeological report, the groundwater table lies 2.6–4.5 m below the surface of the ground (varying with climate and season). That is, the groundwater table is so high that it will inevitably have an impact on the excavation. Dewatering wells are therefore used in the pit to lower the groundwater table to a level below the excavation surface, so that excavation can proceed under dry conditions.

**Figure 5:**
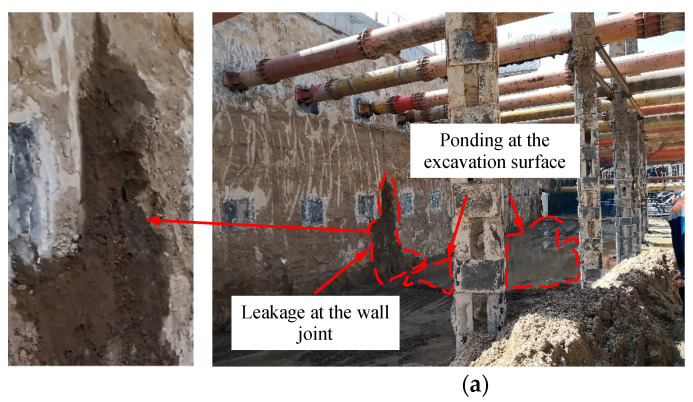

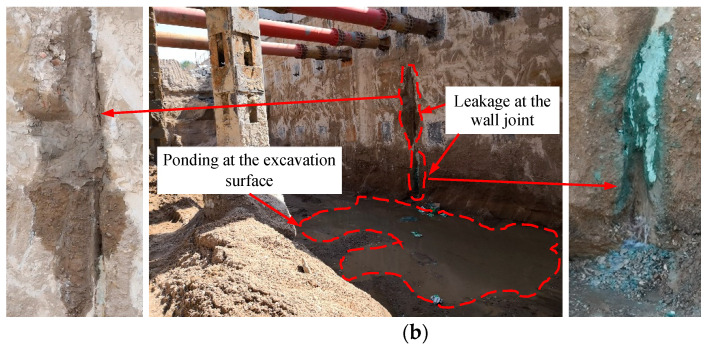
Photographs showing water leakage occurring in the wall joints at the site. The leaks were observed in: (**a**) the east side wall, and (**b**) the west side wall.

The local environment around the excavation is complex; and a welfare institute, service station, and temporary road for vehicles all lie adjacent to the excavation, as shown in Figure 2. Therefore, if the D-wall joints were to leak and result in land subsidence, it is highly likely that damage will be caused to the nearby buildings; there is even the possibility that a geologic disaster could happen. The construction site of the project was investigated prior to the installation of the LDS-DWJ. It was found that there were several groundwater leakage problems in the joints of the D-walls in the sections where excavation had been completed (Zone A in Figure 2) that were clearly visible. In particular, there was some ponding of water on the surface of the excavation, which constituted a serious threat to the safety of the excavation and surrounding buildings (Figure 5). Consequently, it is deemed necessary to find the potential leakage sites in the D-wall joints prior to excavation and apply appropriate mitigating measures in a timely manner to prevent the situation from getting worse.

### 3.2. Design and Installation of the LDS-DWJ

#### 3.2.1. Selection of Detection Points

The location of the pit intended for the pumping station in Zone B (had not yet been excavated) is highlighted in Figure 2. The local depth of the excavation is 15 m at Point L1, while it is 10 m at Point L2). There is a temporary road to the west side of the pumping station carrying lots of vehicles (it is, in fact, one of the main roads connecting north and south Hohhot). If the D-walls in this area were to have defects in their joints, then leakage could occur with water inrush and (possible) piping during excavation. This could result in land subsidence and lead to disaster; it would certainly have an adverse effect on traffic. In addition, the workers in the pit and people walking on the temporary road would also be put in danger. Therefore, the LDS-DWJ was applied in this area to detect potential leaks in the joints of the D-walls.

**Figure 6:**
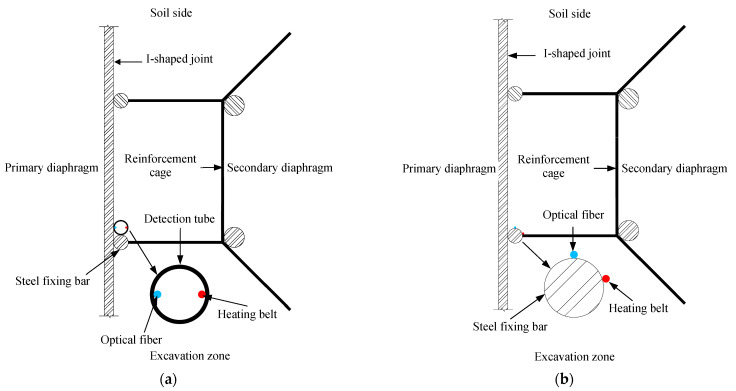
Top views of the test configurations considered in this work: (**a**) detection system incorporating a detection tube (i.e., schemes I; (**b**) tubeless detection system (scheme II).

As mentioned above, detection tubes are used in the system to hold and protect the optical fibers and heating cables; they also act as water barriers to prevent the surrounding groundwater from flowing in, so that optical fibers and heating cables inside work under dry conditions. In addition, detection tubes act as heat conductors. In order to verify the effect of the detection tube, two schemes were designed in this work:

(I) Tubes made of stainless steel: the optical fibers containing FBG sensors and the heating belt were pasted onto the inside surface of the tube, which was then arranged vertically along the D-wall joint (Figure 6a). A steel fixing bar was welded to the end of the steel reinforcement cage and used to fix the position of the detection tube. The detection tube was installed on the inner surface of the steel fixing bar to prevent it from being damaged by collisions occurring between the primary D-wall and side wall of the trench as the steel reinforcement cage was put into the trench.

(II) A tubeless configuration: in this scheme, the optical fiber with FBG sensors and the heating belt were directly attached to the steel fixing bar. The relative positions of the optical fiber, heating belt, and steel reinforcement cage are shown in Figure 6b. As in the other scheme, the optical fiber and heating belt were installed on the inner surface of the fixing bar to prevent them from being damaged when the steel reinforcement cage was put into the trench.

The two schemes were employed at the two positions labeled L1, L2 in Figure 2, respectively. It should be noted that the form of the LDS-DWJ was designed according to the I-shaped metal sheets used in this project. However, this system is not restricted to this kind of water-tight joints. The configuration of the LDS-DWJ (e.g., the length, connection mode and installation position of detection tubes, etc.) can be redesigned to accommodate different kinds of water-stops on the ends of the D-wall panels.

#### 3.2.2. Specific Details of the LDS-DWJ Used in This Project

**Figure 7:**
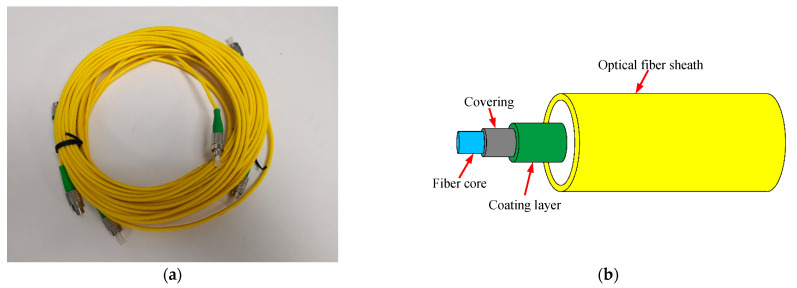
Optical fiber mounted with FBG sensors used in this work: (**a**) real product; (**b**) schematic diagram.

The FBG sensors (DH-Photoelectric Co. Ltd., Jinan, China) used in the system is shown in Figure 7a. These sensors were mounted inside an optical-fiber core, and then wrapped by covering and coating layer in turn. The optical fiber was suspended in the fiber sheath in order to eliminate the influence of stress on the sensors, as shown in Figure 7b. According to the data provided by the manufacturer, the main operating parameters of the sensors are as follows: the operating temperature ranges from −40 to 120 °C; precision is ±0.5 °C; and sensitivity is 10 pm/°C. The FBG sensors were equally spaced over the depth range of the joints, thus covering the whole of the D-wall and detecting all of the potential leak sites in the joints. According to the description given above, the D-wall is 26 m deep at the detection points L1 and L2 (the wall is 25 m high and there is a capping beam of height 1 m on top). The sensors were arranged from the top to the bottom of the D-wall, ranging from a depth of 1 to 26 m below ground level (GL). Thus, there were 51 FBG sensors assigned at each detection point, with an inter-sensor spacing of 0.5 m.

According to the positions of the sensors, the top–down temperature distributions along the detection tubes (steel fixing bar at L2) placed alongside the joints can be obtained. However, the reliability of the method is relatively low if all 51 FBG sensors are mounted inside a single optical fiber simply because the failure of just one FBG sensor at a certain point in the fiber can lead to signal blocking. As a result, the demodulator will not receive signals from sensors that lie beyond the damaged one.

To address this problem, the FBG sensors were spread over 3 parallel optical fibers (17 in each fiber). The sensors were still spaced apart by 0.5 m but were offset between fibers giving a staggered arrangement of sensors that together gave a continuous coverage of the full length of the 25-m-deep wall (from the top of the D-wall itself to the bottom). In this way, the reliability of the thermometric subsystem was significantly improved.

**Figure 8:**
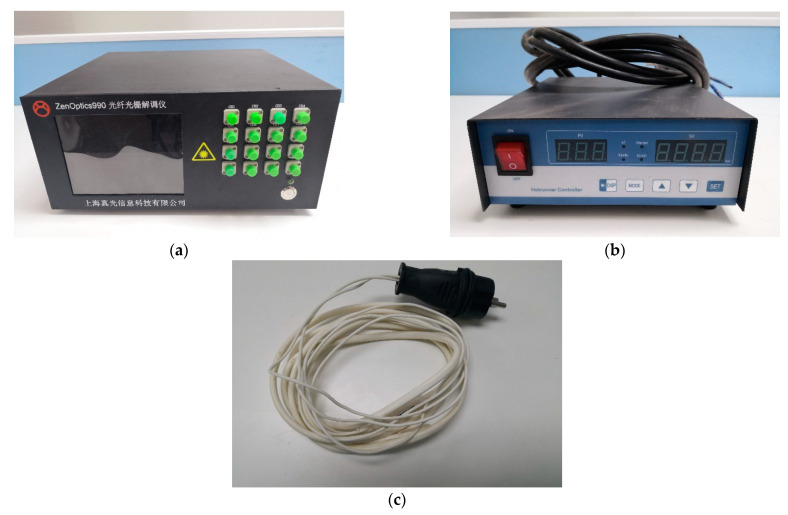
Equipment used in this work: (**a**) the FBG demodulator; (**b**) the thermostat; (**c**) the heating belt.

The FBG demodulator (Zen Optics Co. Ltd., Shanghai, China) used in the system is shown in Figure 8a. This device has a total of 16 channels, a precision of 1 pm, and resolution of 0.1 pm (according to the manufacturer).

Two thermostats (model HRTC-02F; Figure 8b), manufactured by Tinko Instrument Co. Ltd. (Suzhou, China), were used in the experiments, one for each of the measurement locations L1 and L2. These devices monitor the temperature of a control point using a thermocouple and adjust the current to their respective heat belts accordingly to maintain the temperature at a preset value, with a resolution of ±0.2 °C (according to the manufacturer). Two heating belts were assigned to each detection point to ensure their reliability and heating efficiency. The effective length of each heating belt was 25 m (Figure 8c) and their power rating was 40 W/m.

**Figure 9:**
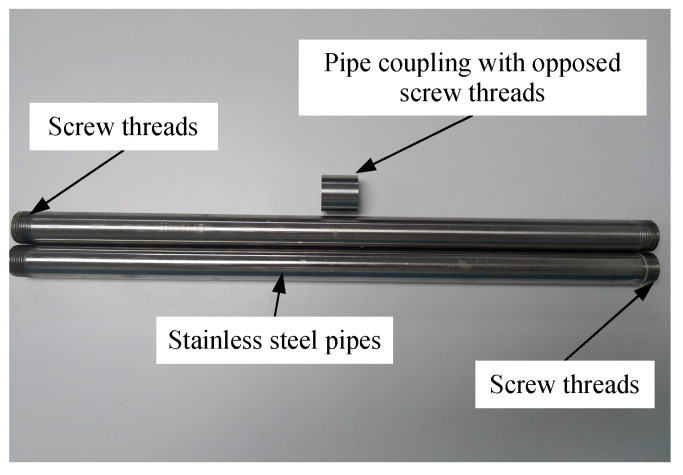
Stainless-steel detection tubes and their coupling mechanism.

The detection tube at L1 was made of 26 stainless-steel pipes, each 1 m in length and 25 mm in diameter with a wall thickness of 1.5 mm. Both ends of each pipe have screw threads cut onto their external surfaces to allow adjacent pipes to be connected together. This is accomplished using coupling tubes that are internally threaded with screw threads that are opposite to but otherwise identical to those on the 1 m pipes (Figure 9).

#### 3.2.3. Installation of the LDS-DWJ

The installation of one of the leakage detection systems typically includes installing the optical fibers with FBG sensors, heating belts, and thermocouples, and then connecting the detection tubes (at L1) to the reinforcement cage. Due to the use of different detection tube designs at the two detection points, the installation procedures are somewhat different in each case. The specific installation processes are outlined below.

##### Point L1 (Stainless-Steel Detection Tube)

The FBG sensors and heating belts were first checked to verify that they worked normally. Thereafter, the three optical fibers were laid out in parallel on flat ground and staggered to make sure the parts mounted with FBG sensors formed a continuous arrangement of sensors covering a length of 25 m. Then, the two heating belts were placed together with the three optical fibers. When all five cables were ready (three optical fibers and two heating belts), the bottom ends of the cables were inserted into one of the steel pipes (the one to be installed at the bottom of the D-wall). The cables were then attached to the inner surface of the steel pipe using cyanoacrylate glue, according to the configuration shown in Figure 6a. A water-stop was then attached to the bottom end of the pipe to stop groundwater entering into the tube.

**Figure 10:**
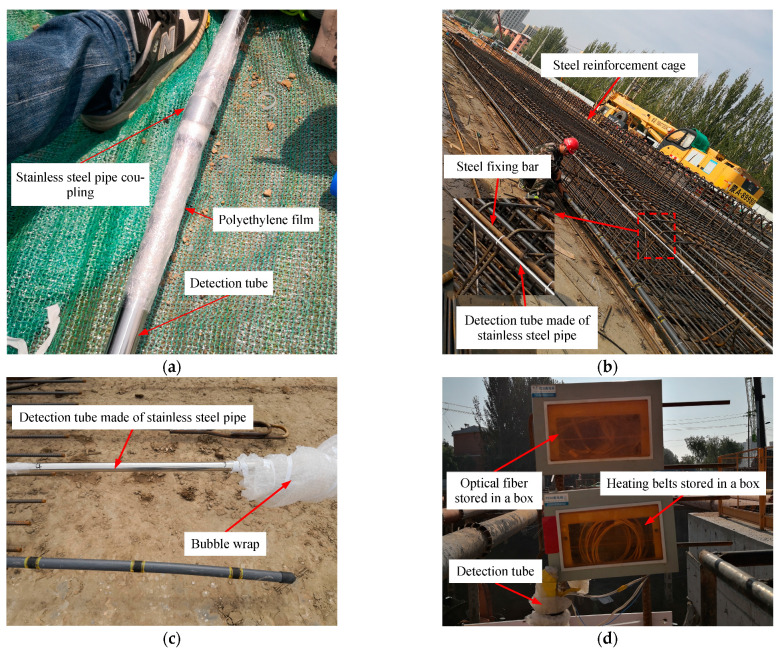
Photographs showing some of the installed parts of the detection system at L1: (**a**) a coupling tube between two adjacent short pipes wrapped in polyethylene (PE) film; (**b**) the detection tube fixed to the reinforcement cage; (**c**) cables outside the detection tube protected by bubble wrap; (**d**) detection point L1 after the installation of the facilities had been finished.

Thereafter, the cables were inserted into the rest of the steel pipes one by one. The optical fibers and heating belts should be glued to the inner surface of the steel pipe as each section of pipe were installed. The thermocouple was attached to the heating belts before the last two steel pipes were installed (so its location corresponded to 1 m below the top of the wall or, equivalently, 2 m below GL when construction of the D-wall was finished). It should be noted that every connection between adjacent pipes (via the coupling tubes) was tightly wrapped in polyethylene (PE) film to ensure that groundwater could not seep into the detection tube (Figure 10a).

When the detection tube was fully assembled, it was fixed to the steel fixing bar (welded to the end of the steel reinforcement cage of the secondary diaphragm), as shown in Figure 10b. The FBG sensors and heating belts were then checked once more to verify that they were still working normally. The parts of the cables outside the detection tube were then wrapped in bubble wrap to protect them from being damaged in subsequent work (e.g., when the steel reinforcement cage was lowered into the trench or when concrete casting was carried out), as shown in Figure 10c.

After all of the work outlined above was finished, a crane was used to lift and lower the reinforcement cage/detection tube assembly into the trench. It should be noted that the area close to the interface between the concrete and I-beam is the main area where defects (and therefore leaks) may occur. Consequently, the position of the reinforcement cage must be adjusted as it is being lowered into the trench so that the detection tube clings to the surface of the water-stop (I-beam) at the primary diaphragm end.

Finally, concrete was poured into the trench. During this process, care was taken to ensure that the optical fibers and heating belts were protected. Figure 10d shows the detection point L1 after the facilities had been installed and construction of the wall finished.

##### Point L2 (Tubeless Installation) 

**Figure 11:**
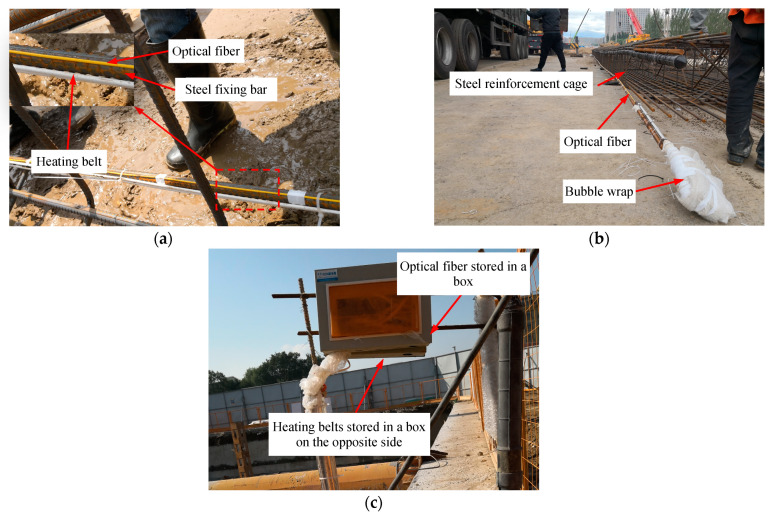
Installation of facilities at L2: (**a**) optical fibers and heating belts fixed onto the reinforcement cage; (**b**) cables outside the detection tube wrapped to protect them; (**c**) detection point L2 after finishing the installation of facilities.

Figure 11 shows the installation of facilities at detection point L2. In this case, the optical fibers with FBG sensors and heating belts were directly attached to the surface of the steel fixing bar without the use of a detection tube. As before, the FBG sensors and heating belts were checked to ensure that they worked normally before installation. Thereafter, the three optical fibers were staggered to make sure they formed a continuous array of FBG sensors covering a length of 25 m. They were then attached to the steel fixing bar in accordance with the position shown in Figure 6b. Figure 11a shows the optical fibers and heating belts fixed onto the reinforcement cage. The thermocouple was again attached to the heating belt 1 m below the top of the wall (2 m below GL after the D-wall had been finished).

After finishing the installation, the FBG sensors and heating belts were checked to ensure that they worked normally. Then, the rest of the cables not attached to the steel fixing bar were bubble-wrapped to protect them from damage in subsequent work (Figure 11b). The remaining installation steps followed those used at L1. Figure 11c shows detection point L2 after finishing the installation of facilities and construction of the D-wall.

### 3.3. In Situ Detection and Monitoring

#### 3.3.1. Monitoring Plan

The D-wall was constructed in the pumping station zone from 20 June to 8 July 2019. Leakage detection and monitoring was performed once a day from 7 July to 21 July before the soil was excavated from the pit (the soil in the pumping station zone was excavated from 29 July to 10 August). It should be noted that monitoring was not performed on some days due to construction activity (hence some data are missing in the figures presented in the following section).

#### 3.3.2. Monitoring Procedure

The temperature distribution along the detection tube (fixing bar at L2) was monitored twice at each monitoring time to find: (i) the initial temperature distribution, and (ii) the temperature distribution when the temperature of the control point rises to a preset value (60 °C in this instance). In order to carry out leakage detection, certain devices needed to be installed (Step 1) before the temperature monitoring procedures (Steps 2–4) could be performed. The details of the operating procedures are as follows.

**Step 1:** Device installation. The optical fibers were connected from the two detection points to the demodulator. Then, the heating belts and thermocouples were connected to the corresponding channels of their thermostats.

**Step 2:** Initial temperature distribution. The FBG demodulator was turned on. The wavelength shift of the light at ambient temperature was monitored and recorded over a period of 5 min.

**Step 3:** Artificially-raised temperature distribution. The thermostats were turned on, and the target temperatures of the two control points were set to 60 °C at L1 and L2, respectively. The heating process took about 30 min. During this time, the FBG demodulator continuously recorded the wavelength shift of the light.

**Step 4:** Saving the recorded data. The data that had been recorded by the FBG demodulator were saved. The demodulator and thermostats were then turned off and tidied up. The cables were then put away to finish the monitoring process.

## 4. Results and Discussion

When the temperature of an FBG sensor changes, there is a shift in the Bragg wavelength *λ_B_* of the FBG [15,27,29,30]. The relationship between the temperature change, ΔT, and shift in Bragg wavelength, $\Delta \lambda_{B}$, can be expressed in the form:

(1) $$\Delta \lambda_{B}=k\Delta T=\left(\alpha +\delta \right)\Delta T,$$

where α is the coefficient of thermal expansion, δ the thermo-optic coefficient of the fiber material, and *k* is the thermal coefficient (given by *k* = *α* + *δ*), so that

(2) $$\Delta T=\Delta \lambda_{B}/k,$$

This expression can be used to determine the temperature of the detection tubes (fixing bar at L2) at the points where the FGB sensors are located. Thus, the temperature variation along the lengths of the joints at each detection point can be obtained as a function of depth.

**Figure 12:**
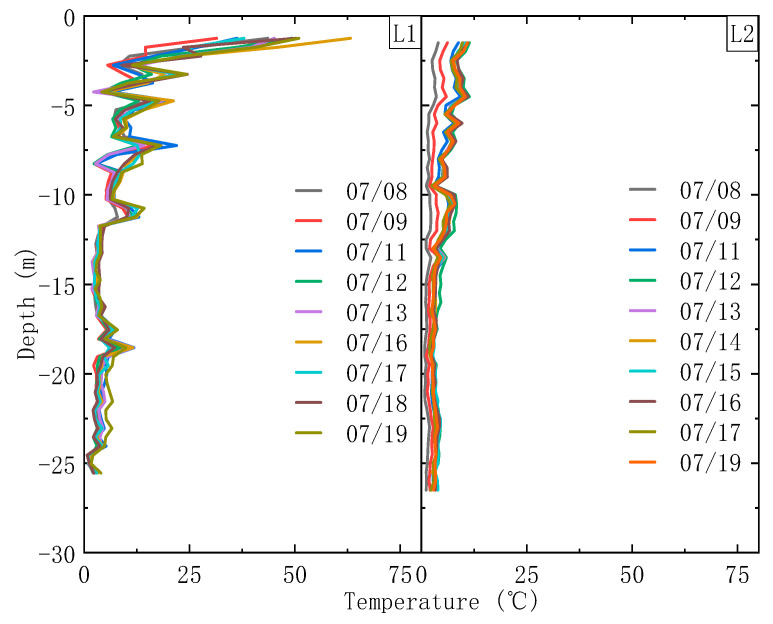
Temperature profile along the joint at L1 and L2 after active heating.

The actual depths of the FBG sensors deviated slightly from their design depths, due to the fact that there was a certain variation in the exact position of the wall. Therefore, the temperature distributions presented in this section are related to the actual depth of the wall, according to the actual wall elevation and the position of the FBG sensors relative to it, as shown in Figure 12. It should also be noted that the temperature distributions in this section correspond to the temperature increments produced by the heating subsystems.

### 4.1. Point L1

Figure 12 illustrates the temperature distributions measured at L1 on different days before the pit was excavated. As can be seen, the highest temperatures are measured upon heating at the top of the wall. The temperature then decreases rapidly with depth down to a depth of about 13 m. The temperatures below 13 m are clearly much lower than those above. This general variation can be explained in terms of the initial temperature distribution in the strata which gradually decreases with depth. Therefore, the heat supplied to the lower parts of the detection tube is dissipated relatively quickly so that the temperature increases in the deeper parts of the strata are smaller. Moreover, the presence of groundwater in the strata will act as a heat sink and will tend to keep the temperature in the joint areas of the deep parts of the wall constant (depths below 13 m).

It can also be seen from Figure 12 that the temperatures recorded at the same depth are different on different dates. This is because a great deal of heat is produced by the concrete during the hydration process which causes the temperature to change over time [31,32,33,34]. This will affect the temperature increment produced by the heating belt in the joint area.

It is notable that the temperatures at 2.75, 4.25, 6.75, and 10.25 m below the ground level are significantly lower than those of nearby areas. The temperatures in the depth range 11.75–15.75 m and 19.05–25.55 m are also slightly lower than those in the area around 17.55 m. Therefore, there appear to be hydraulic connections between the groundwater in front of and behind the D-wall at these locations, due to the presence of defects (leakage will occur where the joint is defective and this will take heat away from the detection tube more quickly, resulting in a lower temperature around the defect compared to other, non-defective, zones). It can also be notable that temperature suddenly drops at 8.25 m at some times. This suggests that the defects at this position may not exist or be repaired due to bentonite inclusions or slightly decomposed concrete of lower strength. Their occurrence in the joints prevents water inflow through the joints. However, this situation may change instantaneously when the water pressure reaches a certain value that causes damage of the inclusions and slightly decomposed concrete [16].

**Figure 13:**
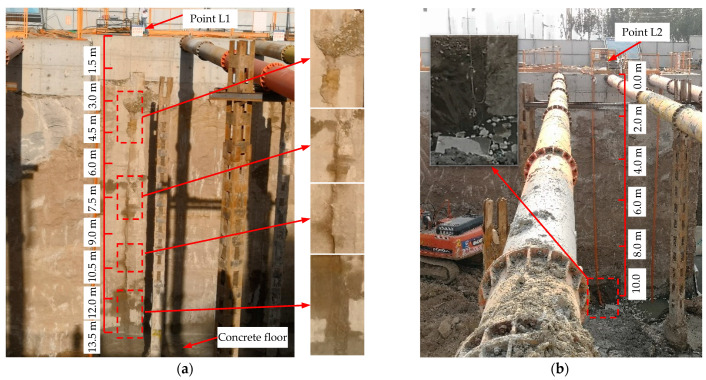
Leakage occurring in the wall joint after finishing excavation. (**a**) leakage occurring at L1; (**b**) leakage occurring at L2.

Figure 13a shows the leakage observed at L1 after the excavation of the pit was finished and the casting of the concrete floor (1.5 m thick) was completed in the pumping station on August 13th. Several water leaks, including leakage at 8.25 m (meaning that inclusions or slightly decomposed concrete in the defect are washed away by water inflow after excavation), can be seen in the figure occurring at the joint between the two wall panels above the concrete floor (13.5 m below ground level). Table 1 summarizes a detailed comparison of leakages between predictions and field observations. The comparison suggests that the locations of these leaks are consistent with the detection results at L1. The detection results of the points below the bottom cannot be verified because over-excavation was forbidden. But, it can still be concluded that the LDS-DWJ using a stainless-steel detection pipe can effectively detect the potential leakages.

### 4.2. Point L2

Figure 12 also presents the temperature distributions recorded at L2 on different days before pit excavation. The general trends at L2 are essentially the same as those observed at L1 and for the same reasons. Once again, the temperature falls from the top of the wall to a depth of about 13 m, and the temperature below this point is lower than that above. Furthermore, the temperatures of the deeper areas (below 13 m) once again vary very little and the temperature at a given depth varied on different days due to heat generated by the hydration of the concrete (as it did at L1).

It can also be seen from Figure 12 that the temperature suddenly drops at the points 5.5 m, 6.5 m, 8 m, 9.5 m, and 13 m below the ground level. This suggests (according to the operating principles of the system outlined above) that there is probably a leak occurring in the joint at this depth.

Figure 13b is a photograph of the leakage observed at L2 after the pit was excavated. It can be seen from the picture that water infiltration is occurring 9.5 m below the surface of the ground, indicating that leakage is occurring in this area. This is again consistent with the detection results. However, there is no leakage or water infiltration occurring at 5.5 m, 6.5 m, or 8.0 m depths, which is inconsistent with the detection results. Consequently, the detection results based on the LDS-DWJ using the tubeless configuration are unreliable, as summarized in Table 1. 

### 4.3. Discussion

The measurement results from L1 (stainless-steel detection tube) and L2 (no detection tube) are somewhat different, showing that the detection scheme used does make a difference.

First of all, the temperature profile at L1 is higher than that at L2. This is because the stainless-steel detection tube has a ‘wrapping effect’, which makes the heating belt at L1 slightly more efficient than that at L2. In other words, all of the heat from the heating belt is transferred by conduction through the stainless-steel tube, and can be measured by the FBG sensors that are attached to the inner surface of the steel pipe at Point L1. As for L2, however, some of the heat from the heating belt is diffused into the surrounding environment through the concrete, while only part of the heat is transferred through the steel fixing bar, resulting in a smaller temperature increment in it. Thus, the temperature measured by the FBG sensors is lower at L2 than that at L1.

It can also be seen from Figure 12 that the temperature distribution curve fluctuates significantly. The primary reason for this is that there is leakage occurring in several places at L1. Furthermore, uneven heating by the heating belts also causes the temperature distribution curve fluctuating along the D-wall joints at both of the detection points.

The temperature distributions at L1 and L2 show that the temperature along a joint decreases as depth increases. This implies that the ambient temperature affects the heating effect produced by the heating belts. Therefore, it is reasonable to suggest that water leakage through defects should be detected by the temperature drop they cause in the nearby area. However, the heating effect produced by the heating belts is very low once a certain depth is reached, which makes the technique rather insensitive to leakage in D-walls that are deep. Therefore, materials with higher heating efficiencies should be used to heat the detection tubes.

This is the first time that this detection system has been applied to a real project. Naturally, there are some differences between the systems used in the field application and those used in the indoor tests carried out (length of the detection tube, installation procedures, ambient temperature, etc.). Therefore, the detection system should be further optimized in the future to make it more suitable for use in the harsh environments encountered in construction sites. First of all, the structure, strength, and stiffness of the detection tube should be optimized. This will ensure that the optical fibers and heating belts can be quickly installed without being damaged and make operation of the system more convenient for the on-site workers. Secondly, the effect of low underground temperatures on the heating effect produced in long detection tubes should be studied in greater detail.

## 5. Conclusions

A new leakage detection system for D-walls has been applied to a project site in Hohhot, China. The following conclusions can be drawn based on the results from the field application:

(1) The proposed detection system is based on FBG sensing technology. The successful application of the system in the field verifies its feasibility and effectiveness at determining the occurrence and positions of leaks in a D-wall. The system monitors the temperature distribution along the wall joint and allows leaks to be located according to their effect on the temperature distribution (a sudden drop in temperature implying the presence of a leak).
(2) A detection tube made of stainless steel is strong enough to protect the FBG sensors and heating belts from damage. A steel detection tube can also conduct heat efficiently. This makes it sensitive to changes in ambient temperature and these changes can be readily detected by FBG sensors glued onto the tube’s inner surface.
(3) Installation is simple and efficient if the optical fibers and heating belts are directly fixed onto the steel reinforcement cage. However, the thermal energy from the heating belt is diffused more rapidly when it is directly exposed to the concrete and reinforced cage. In this case, there is limited scope for improving the temperature field in the area of the joint. This means that this detection scheme is unlikely to be sensitive enough to detect potential leaks, and even provides inaccurate prediction.

It should be mentioned here that the work presented in this paper does have its limitations. The major issue is that uneven heating by the heating belts occurs, which may influence the monitoring results. The leakage detection is performed in sunny days; it is uncertain whether different weather, such as rainfall, can affect the monitoring results. In follow-up studies, therefore, it is intended that more extensive laboratory and field tests will be conducted to improve this situation.

## Figures and Tables

**Table 1:** Comparison of leakages between predictions and field observations.

C	Position (m)	Predicted Result	Field Observation
L1	2.75	Yes	Yes
	4.25	Yes	Yes
	6.75	Yes	Yes
	8.25	Yes	Yes
	10.25	Yes	Yes
	11.75–13.5	Yes	Yes
L2	5.5	Yes	No
	6.5	Yes	No
	8.0	Yes	No
	9.5	Yes	Yes

## Data Availability

The data presented in this study are available on request from the corresponding author.
